# “Get the best out of what comes in” – adaptation of the microbiota of chamois (*Rupicapra rupicapra*) to seasonal forage availability in the Bavarian Alps

**DOI:** 10.3389/fmicb.2023.1238744

**Published:** 2023-10-02

**Authors:** Sarah-Alica Dahl, Jana Seifert, Amélia Camarinha-Silva, Angélica Hernández-Arriaga, Wilhelm Windisch, Andreas König

**Affiliations:** ^1^Wildlife Biology and Management Unit, Chair of Animal Nutrition and Metabolism, Technical University of Munich, Freising, Germany; ^2^HoLMiR – Hohenheim Center for Livestock Microbiome Research, University of Hohenheim, Stuttgart, Germany; ^3^Institute of Animal Science, University of Hohenheim, Stuttgart, Germany; ^4^TUM School of Life Sciences, Technical University of Munich, Freising, Germany

**Keywords:** chamois, *Rupicapra rupicapra*, microbiota, rumen content, bacteria, season

## Abstract

As an inhabitant of the Alps, chamois are exposed to significant climatic changes throughout the year and are also strongly confronted with changing forage availability. Besides horizontal and vertical migratory movements as an adaptation, it undergoes physiological transformations and dynamic changes in the ruminal microbiota.

The following study used 48 chamois of different ages and genders to investigate to which extent the ingested food plants, the resulting crude nutrients in the rumen (reticulorumen) contents, and the bacterial microbiota in the rumen and their fermentation products were influenced by the changes over the seasons. Very little is known about the microbiota of wild ruminants, and many bacterial taxa could only be determined to certain taxonomic levels in this study. However, adapted microbiota reflects the significant changes in the ingested forage and the resulting crude nutrients. For some taxa, our results indicated potential functional relationships. In addition, 15 genera were identified, representing almost 90% of the relative abundance, forming the central part of the microbial community throughout the year. The successful and flexible adaptation of chamois is reflected in the chamois rumen’s nutrient and microbial profile. This is also the first study that analyzes the microbiota of the chamois using rumen samples and considers the microbiota in a seasonal comparison.

## 1. Introduction

The chamois (*Rupicapra rupicapra*) is a wild ruminant species that successfully adapts to conditions in extreme habitats. It is the character species of the Bavarian Alps. But even though recreational users of the Alps highly value it, we know very little about the status of the Bavarian population. Therefore, it has been the focus of some emotionally driven discussions for years. These discussions have triggered several new research projects, such as those to determine population sizes and space use. Apart from the need for knowledge about the space use and population size of the animals in the Alpine region, it is crucial to know how the population is doing, what food is available to it, what it uses, what seasonal fluctuations it faces, and how can it successfully adapt to them.

Like all inhabitants of the Alps, chamois are sometimes confronted with extreme seasonal and spatial differences in weather conditions or forage availability (Espunyes et al., 2019a). Thus, the chamois’ choice of location during the year and, therefore, the availability of forage is strongly dependent on the photoperiod. Hard winters with extreme snow conditions force the animals to use their energy reserves very sparingly. And not only the availability of plants but also their nutrient composition changes significantly over the year.

The vegetation period, which lasts on average 110–130 days in the Alps (approx. June–October) (Kölling et al., 2005), often starts at the middle or end of May, depending on the altitude and plant species. For every 100 meters of altitude, the phenophase is delayed by an average of 3.7 days (Cornelius et al., 2013). And the influence of climate change is already impacting the duration and starting time (Linderholm, 2006). Chamois increasingly move to the high plateaus during the summer and autumn months. Alpine lawns are used as roosts almost all year round, but more so in winter and spring. Most chamois migrate to lower altitudes in winter in the forest and mountain pine scrub areas. At times, sun-exposed high locations in the rocky area are also used (Schröder, 1971).

The chamois is a perfect example of the browser-grazer-continuum (Pérez-Barbería et al., 2004). It is classified as an intermediate feeding type (Hofmann, 1989). But over the year, chamois show a fluid change between the food niches of browsers and grazers. Chamois from the European Alpine populations consume high proportions of grasses (Schröder, 1977; Trutmann, 2009; Andreoli et al., 2016). A focus on woody plants is also known from other countries (Yockney and Hickling, 2000). These proportions of the individual plant groups consumed vary greatly depending on habitat and season (Bertolino et al., 2009; Trutmann, 2009; Corlatti et al., 2022).

The most crucial tool ruminants have to effectively adapt to changing forage availability is their rumen microbiome. It is essential for converting plant food into usable volatile fatty acids, which serve as a primary source of energy (Krause et al., 2003; Owens and Basalan, 2016). In contrast to domesticated ruminants, very little is known about wild ruminants and the related microbiome in the digestive tract. For a few wild ruminants, there are now a handful of studies, e.g., roe deer, moose, or reindeer (Sundset et al., 2007; Ishaq and Wright, 2012; Pope et al., 2012; Ricci et al., 2019). For chamois, to the best of the authors’ knowledge, there is currently only one study available (Smoglica et al., 2022), which takes into account the latest methods of genome sequencing (fecal samples, n = 5). Closer research would, therefore, contribute to the Bavarian Alpine region and provide new insights into the species.

The present analysis intends to show how the adaptations occur at the forage intake, nutrient, and microbiota levels. Besides the analysis of crude nutrients, fermentation products, and botanical rumen content analysis, the main focus is the description of the rumen’s bacterial microbiota and its most abundant representatives. Therefore, 48 chamois reticulorumen samples from Bavarian alpine habitats were analyzed.

## 2. Materials and methods

### 2.1. Sample material and study area

The chamois samples were collected in 2017–2019 from forestry operations in the Bavarian (Germany) Alps (Ruhpolding 47.722929 12.333739, Bad Tölz 47.516315 11.354989, and Berchtesgaden 47.654045 12.823182), which are managed by the Bayerische Staatsforsten AöR (Bavarian Forestry Authority, a public-law institution). The samples came from various regions and altitudes, where the climate can often differ significantly on a tiny scale. The sample sites are located in the submontane to high subalpine/alpine level and are characterized by a pre-alpine climate. The annual mean temperature is 5.1–5.4°C, and the mean temperature in the growing season is 11–15°C. The average precipitation is 1700 to over 2000 mm. On average, there are 150 snow days per year, and the growing season usually lasts between 110 and 130 days and starts in mid to late May (depending on altitude and plant species). Characteristic vegetation types are mixed mountain forests, spruce forests, mountain pine bushes, and alpine lawns (Kölling et al., 2005; Cornelius et al., 2013).

All chamois samples were obtained within the regular hunting bags. As the samples originate from redevelopment areas for rehabilitating protection forests, closed seasons had already been lifted, based on the ordinance on changing the hunting seasons for hoofed game in redevelopment areas in the administrative district of Upper Bavaria of 14 February 2014. Thus, year-round sampling was guaranteed. The samples (reticulorumen with content and other organs) were frozen at −18°C as soon as possible after collection. Furthermore, the condition and constitution data of each animal were recorded. The age was determined based on counting the horn rings, the tooth development, and the tooth section method (Mitchell, 1967) and divided into age classes (juvenile, subadult, adult). For this microbiota study, 48 animals were sampled between September 2017 and March 2020. Supplementary Figure S1 gives information regarding botanical rumen content analyses.

The samples were thawed for further processing in the laboratory, and the rumen (reticulorumen, hereinafter only referred to as rumen) was opened for sampling. The rumen contents (solid and liquid) were taken from the rumen and then homogenized by stirring. Appropriate quantities were taken for DNA extraction, crude nutrient analysis (500 mL + 500 mL reserve), and botanical rumen content analysis (250 mL). Afterward, rumen liquid was extracted from the total content to analyze the fermentation products.

### 2.2. Analyses of crude nutrients and fermentation products

The rumen content was centrifuged (4,400× *g*, 15 min, 21°C) to analyze the crude nutrients. The separated solid phase was then freeze-dried and ground (1 mm grain size).

The crude nutrients were analyzed using standard feed analysis procedures (Weender and VanSoest analysis; Methods 3.1, 4.1.1, 5.1.1, 6.1.1, 8.1, 6.5.1, 6.5.2, 6.5.3; VDLUFA, 2012). The contents of crude protein (CP), total lipids (TL), crude ash (CA), acid detergent fiber (ADF), neutral detergent fiber (NDF), and acid detergent lignin (ADL/lignin) were determined analytically. Hemicellulose, cellulose, and non-fiber-carbohydrates (NFC) content were calculated from the difference between the dry masses and the other crude nutrients. NDF is addressed to total fiber in the text. The crude fiber content (*CF*) was additionally analyzed and quoted in Supplementary Table S1 for comparison with older studies.

The rumen liquid extracted for the analysis of the fermentation products was also centrifuged, and the ammonia and lactate content was then determined by photometric measurement at 340 nm (Ammonia test kit: Randox Laboratories Ltd., Crumlin, United Kingdom, Manual AM 1015; lactate test kit: Boehringer Mannheim/R-Biopharm AG, Darmstadt, Germany).

The volatile fatty acids (VFA’s: acetic (AA), propionic (PA), butyric (BA), valeric (VA), isobutyric (IBA), and isovaleric acid (IVA)) in the rumen liquid were analyzed by gas chromatography (chromatograph: Perkin Elmer, Clarus 580, Waltham, Massachusetts; internal standard for calibration: 100 μL of 2-methyl valeric acid, diluted with 250 mL 2% metaphosphoric acid).

### 2.3. DNA extraction and illumina amplicon sequencing

The DNA was extracted from 200 to 250 mg of the homogenized rumen content. The extraction was carried out according to Burbach et al. (2016) and a FastDNA™ SPIN Kit for Soil (MP Biomedical, Solon, OH, USA) were used. The quality and purity of the DNA extracts were checked using the Qubit 2.0 fluorometer (Thermo Fisher Scientific, Waltham, MA, USA).

The V1-2 region of the 16S rRNA gene was targeted, and the amplicon library preparation was performed according to Kaewtapee et al. (2017). Each sample was subjected to two rounds of PCR; some samples required a pre-PCR step (10 cycles) to ensure the correct amplification. The PCR reaction mixture for the 1st PCR (each 20 μL): 4 μL 5x Prime Star buffer (TaKaRa Bio Inc., Kusatsu, Japan), 1.6 μL deoxynucleoside triphosphate mixture, each 0.5 μL primer (1:10 diluted), 0.2 μL PrimeStar HS DNA polymerase (250 U, TaKaRa Bio Inc., Kusatsu, Japan), 1 μL enhancer (BioStab PCR optimizer (II) 53,833-5ML-F, Sigma-Aldrich®, Merck KGaA, Darmstadt, Germany) (only used for PCR 1) and 1 μL template DNA. First PCR conditions: initial temperature of 95°C for 3 min, followed by 15 cycles of denaturation at 98°C for 10 s, annealing at 55°C for 10 s, extension at 72°C for 45 s, final extension at 72°C for 2 min. The second PCR was performed with 1 μL from the first PCR. The procedure corresponds to the first PCR but with 20 cycles. The reaction mixture for the 2nd PCR: 10 μL 5× PrimeStar Buffer, 4 μL dNTP mixture, each 1.25 μL primer (1:10), and 0.5 μL polymerase (50 μL in total). The expected amplicons were confirmed using gel electrophoresis, normalized using SequalPrep™ Normalization Kit (Applied Biosystems), purified using MinElute PCR Purification Kit (Qiagen), and sequenced using 250 bp paired-end sequencing chemistry on Illumina Novaseq 6,000.

MOTHUR (Kozich et al., 2013) v1.44.3 was used for processing the sequences. Raw reads (forward and reverse fastq file) were assembled with make.contigs function. Reads with ambiguous bases, homopolymers (>8), and longer than 354 bp were removed. Sequences were aligned to the silva.seed v1.38.1 (Quast et al., 2013). Chimeras were identified using vsearch (Rognes et al., 2016) and removed from the dataset. Unassigned sequences and the reads from organelles were removed. Sequences were classified using the Bayesian classifier and taxonomy set silva.seed v1.38.1. Reads were clustered at 97% identity into OTUs.

### 2.4. Statistics and data analysis

According to Dhariwal et al. (2017), the default settings of MicrobiomeAnalyst were used to filter the 16S rRNA gene data. All features that only occurred in a single sample contained only zeros or occurred in small numbers in some samples were discarded. A filtered and rarefied dataset was used to calculate the alpha diversity measures; for further data filtering minimum count was set to 4, with a prevalence in 20% of the samples. The low variance filter was set to 10% based on the interquartile range. Data were scaled by total, and it was not transformed or rarefied. After filtering these low abundance and rare occurrence taxa, 1,208 OTUs were left for further analysis.

PRIMER 6 (Version 6.1.16) and PERMANOVA (Version 1.0.6) (PRIMER-E, Quest Research Limited in Auckland, New Zealand), as well as SPSS (IBM SPSS Statistics Version 27.0.1.0), were used for statistical analysis. The normal distributions were determined by the Shapiro–Wilk test.

The Shannon diversity index and Permanova assessed alpha diversity. Differences between habitat, season, age class, and gender were determined using a one-way analysis of similarities (ANOSIM). A principal-coordinate analysis (PCoA) was utilized to ordinate the beta-diversity distances using the Bray-Curtis distance method.

The normal distribution of the data was checked with the Shapiro–Wilk test. The Kruskal-Wallis analysis with posthoc Bonferroni was used for pairwise comparison of the abundance means of taxonomic groups. The linear discriminant analysis effect size (LEfSe) analysis was used here to determine which taxa may be related to occurring seasonal differences in the microbiota. Furthermore, the Spearman-Rho correlation was used to test the relationships between crude nutrients, fermentation products, and microorganisms.

## 3. Results

### 3.1. Crude nutrients and fermentation products

**Figure 1:**
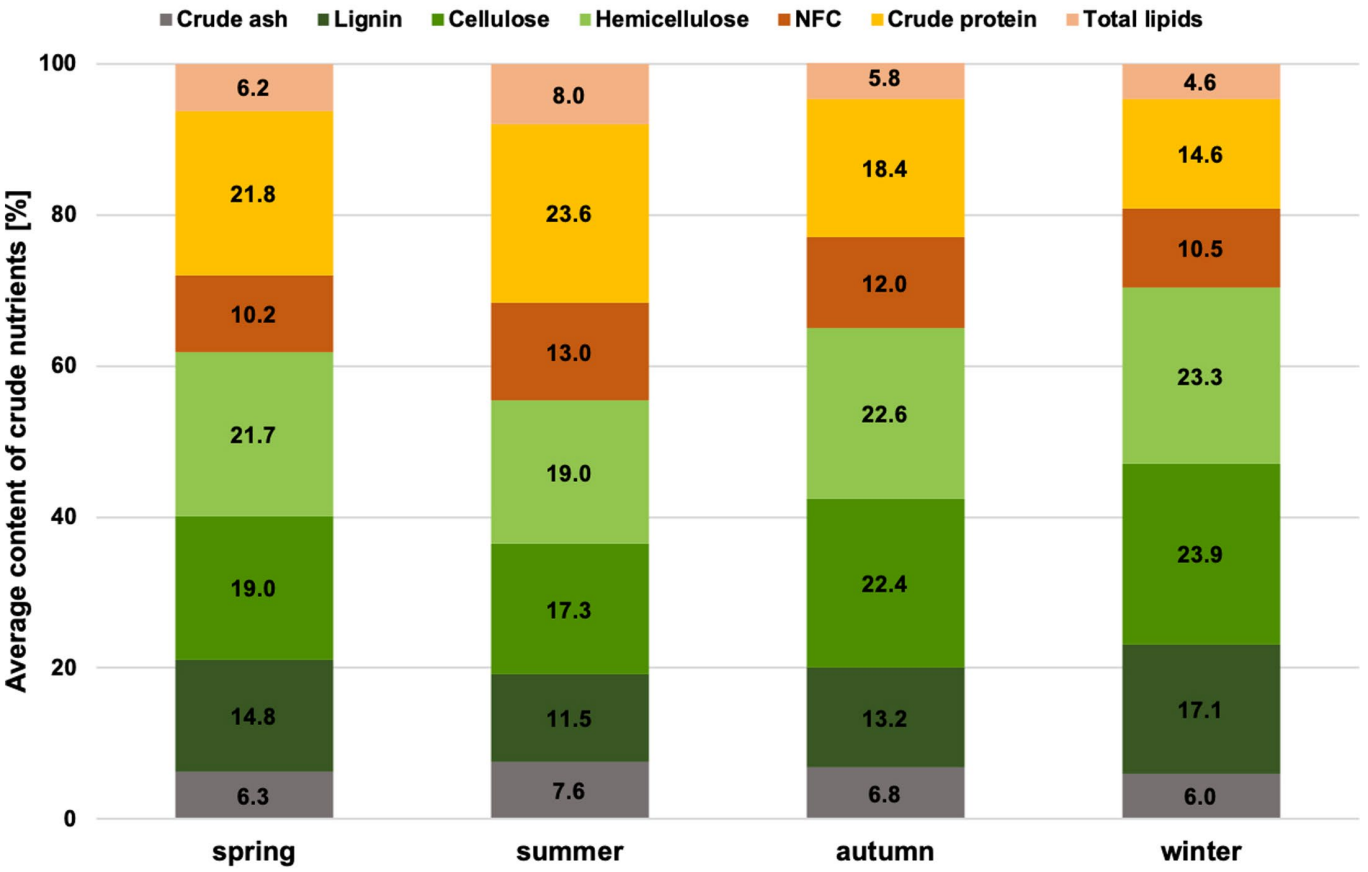
Average crude nutrient contents in chamois rumen content per season.

**Figure 2:**
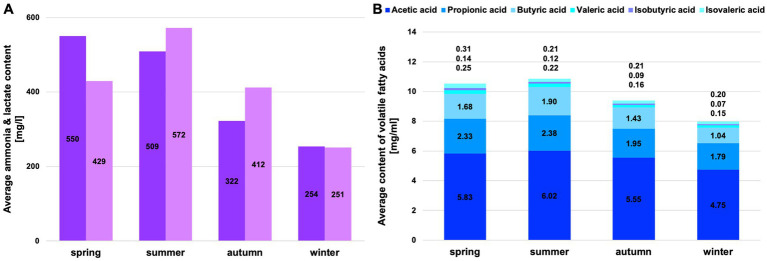
Average content of fermentation products in the rumen liquid. **(A)** Average content of ammonia and lactate in the rumen liquid per season (dark grey = ammonia, light grey = lactate); **(B)** Average content of volatile fatty acids in the rumen liquid per season.

The proportion of crude nutrients (Figure 1) and fermentation products (Figure 2) differs significantly for the factor season (*p* = 0.0002, except hemicellulose, NFC & acetic acid) (Supplementary Table S2). No significant difference could be found for the factors of gender and age class. The proportion of crude protein in the rumen content is highest in summer and lowest in winter. The NFC content has similarly high levels in summer and autumn and lower in winter and spring. In winter, however, the proportion of total fibers and all fiber fractions is highest.

The low concentrations of fermentation products in winter may indicate reduced microbial activity due to altered climatic conditions and reduced food resources. Ammonia, valeric acid, isobutyric acid, and isovaleric acid peak in spring (Figure 2). Lactate, acetic acid, propionic acid, and butyric acid increase in summer (Figure 2).

### 3.2. Microbiota

**Figure 3:**
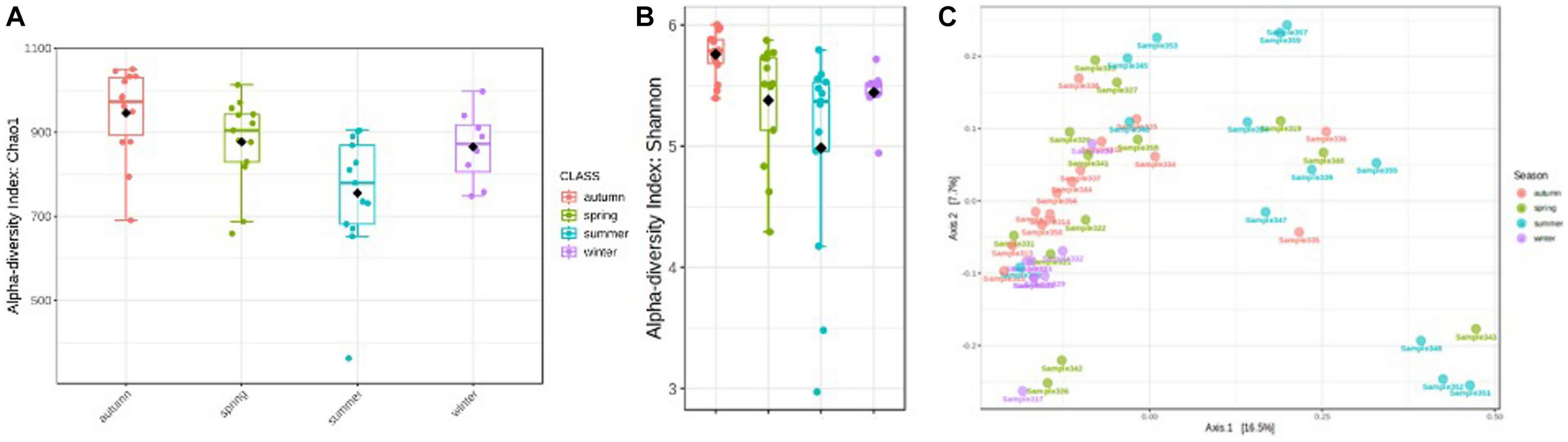
**(A)** Variation of alpha diversity, Chao1 index (*p* < 0.001). **(B)** Variation of alpha diversity, Shannon index (*p* < 0.002). **(C)** Distribution of rumen microbiota samples per season based on OTU level [PERMANOVA test (*p* < 0.001)].

Total read counts varied in the individual samples between 17,000 and 19,600 (Supplementary Table S3). Significant differences in the rumen bacterial community composition were determined for the factor season (*p* = 0.0002). Strong seasonal differences are evident by comparing the diversity measures. The highest ruminal microbial alpha diversity (Chao1 and Shannon) was observed in autumn (Figures 3A,B). The analysis of distribution based on OTU data suggests a higher similarity between rumen microbiota in spring and winter (Figure 3C). A possible explanation for the clustering in the lower right panel is an exceptionally high number of proteolytic taxa compared to the seasonal and annual average (*Prevotella*, *uncl. Prevotellaceae*, and *Fretibaterium*). At the same time, the number of fiber-associated taxa (*uncl. Clostridiales* and *uncl. Ruminococcaceae*) is exceptionally low. A very diverse mixture of taxa, compared to other individual samples, characterizes the samples located in the lower left cluster and could explain this.

A large intersection of OTUs is found for all seasons, which reveals the presence of most OTUs independent of the season throughout the year. Only seven OTUs are absent in summer and 17 OTUs in winter rumen samples.

*Firmicutes* were the most common bacterial phylum in the chamois rumen, averaging 62%. *Bacteroidetes* (approx. 20%), *Actinobacteria* (approx. 14%), and *Synergistetes* (2.4%) occurred with much lower abundance. With an abundance of less than 1%, *Proteobacteria*, *Candidatus* *Saccharibacteria*, *Spirochaetes*, *Tenericutes*, *Planctomycetes*, *Verrucomicrobia*, and SR1 were found in the rumen (Supplementary Table S4). Significant differences for the factor season were already detected at the phylum level. *Bacteroidetes* (*p* = 0.012), *Candidatus Saccharibacteria* (*p* = 0.046), *Firmicutes* (*p* = 0.002), *Planctomycetes* (*p* = 0.022), *Synergistetes* (*p* = 0.005), *Tenericutes* (*p* = 0.008), unclassified *Bacteria* (*p* < 0.001) and unclassified *Saccharibacteria* (*p* = 0.015) differed significantly in abundance between seasons.

**Figure 4:**
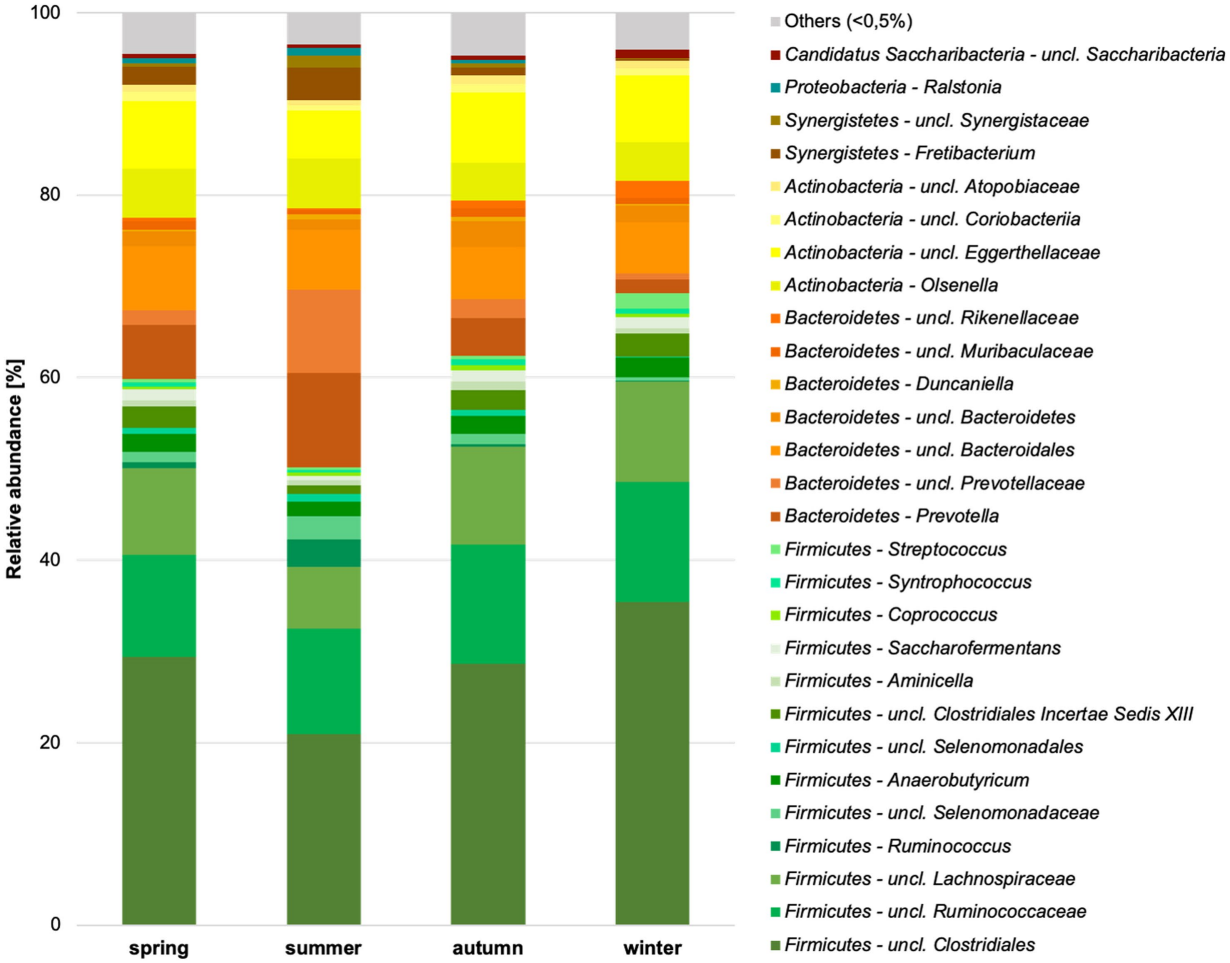
Relative abundance of bacterial genera in the chamois rumen content per season identified based on Silva database. Genera with still undefined genus names are indicated as unclassified (uncl.) with the respective higher taxonomic rank.

Thirty-nine of 73 genera could be classified, and 34 were unclassified at various taxonomic levels (Figure 4; Supplementary Table S5). These 34 unclassified genera corresponded to 78% of the sequences. This suggests an extremely high number of previously unknown genera in the chamois rumen content. There were 39% unclassified sequences at the family level, at the order level at 4.1%, and at the class level at 2.7%. The ratio of *Firmicutes* to *Bacteroidetes* was highest in winter and lowest in summer (spring 3.44, summer 1.83, autumn 3.78, winter 5.68).

**Figure 5:**
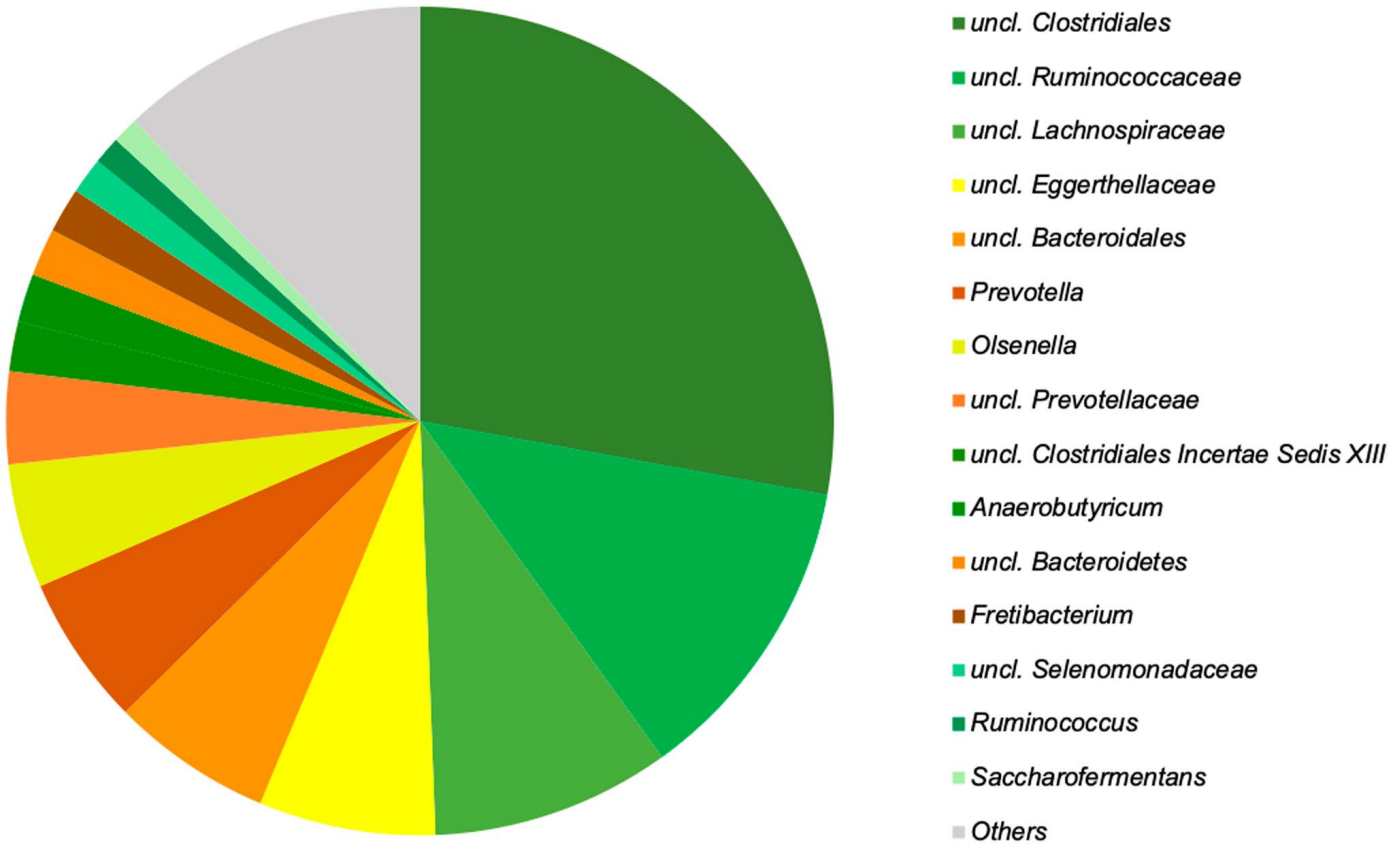
The 15 top genera of the microbiota of chamois rumen, representing 87.8% of the total relative abundance identified based on Silva database. Genera with still undefined genus names are indicated as unclassified (uncl.) with the respective higher taxonomic rank.

At the genus level, 15 genera could be identified, accounting for almost 90% (87.8%) of the relative abundance (Figure 5).

Although these genera are found in high proportions in the rumen at any time of the year, significant seasonal differences can be observed. *Prevotella* occurs significantly more frequently in summer and uncl. *Clostridiales,* as well as uncl. *Lachnospiraceae* in winter. In total, 26 genera show significant seasonal differences (Table 1).

**Table 1:** Genera heatmap showing bacterial genera with significant seasonal differences (white fields = lowest abundance, dark green fields = highest abundance year-round) and their functional mapping (based on literature research).

Genus	Spring	Summer	Autumn	Winter	*p*	Functional mapping	Reference
*Clostridium IV*					0.005	Butyrate producers	Pryde et al. (2002)
*Denitrobacterium*					0.002	Metabolize nitrotoxins and other nitrocompounds	Anderson et al. (2000, 2005)
*Fretibacterium*					0.010	Asaccharolytic, producer of acetic & propionic acid; associated with toxic plant compounds	Vartoukian et al. (2013), Allison et al. (1992)
*Millionella*					0.004	Obesity-promoting, high-fat diet associated	Zhang et al. (2020)
*Paraeggerthella*					0.018	Obesity-promoting, high-fat diet associated	Zhang et al. (2020)
*Prevotella*					0.000	Degradation of protein and peptids as well as xylan and pectin	Purushe et al. (2010)
*Ralstonia*					0.011	Utilize VFA’s & nitrate, denitrification, degrader of plant pathogens	Chakraborty et al. (2009), Schwartz and Friedrich (2001), Zhang and Qiu (2016)
*Ruminococcus*					0.006	Fibrolytic, cellulolytic	Biddle et al. (2013)
*Saccharofermentans*					0.004	Saccharolytic	Chen et al. (2010)
*Selenomonas*					0.007	Degradation of starch and soluble fibres	Scheifinger and Wolin (1973)
*Sporobacter*					0.013	Degradation of aromatic compounds, acetate producers	Grech-Mora et al. (1996)
*Syntrophococcus*					0.046	Demethylisation of syringil lignin	Mosoni et al. (1994)
*uncl. Clostridiales*					0.010	Order of fibre utilizers	Mu et al. (2017), Sharma and Hobson (1985)
*uncl. Clostridiales Incertae Sedis XIII*					0.000	Positive associated with oil addition and low-starch-diet	Zened et al. (2012)
*uncl. Entomoplasmatales*					0.021	Order of taxa associated with pathogenic processes; parasite or symbiont against parasites	Jaenike et al. (2010), Jiggins et al. (2000)
*uncl. Lachnospiraceae*					0.005	Fibrolytic	Biddle et al. (2013)
*uncl. Mollicutes*					0.050	Hydrolyze xylan and starch	Svartström et al. (2017)
*uncl. Prevotellaceae*					0.010	Degradation of protein and peptides as well as xylan and pectin	Purushe et al. (2010)
*uncl. Rikenellaceae*					0.005	Degradation of structural carbohydrates	Pitta et al. (2010)
*uncl. Selenomonadaceae*					0.036	Degradation of starch and soluble carbohydrates	Scheifinger and Wolin (1973)
*uncl. Selenomonadales*					0.042	Degradation of starch and soluble carbohydrates	Scheifinger and Wolin (1973)
*uncl. Synergistaceae*					0.014	Mostly asaccharolytic, degradation of amino acids and proteins, and plant toxins (some individual genera)	Rosenberg et al. (2014), Allison et al. (1992)

Further genera with significant seasonal differences are *uncl.* *Alphaproteobacteria* (*p* = 0.032), *uncl.* *Bacteria* (*p* = 0.001), *uncl. Planctomycetes* (*p* = 0.022) and *uncl. Saccharibacteria* (*p* = 0.004). For these, no clear functional assignment is possible.

**Figure 6:**
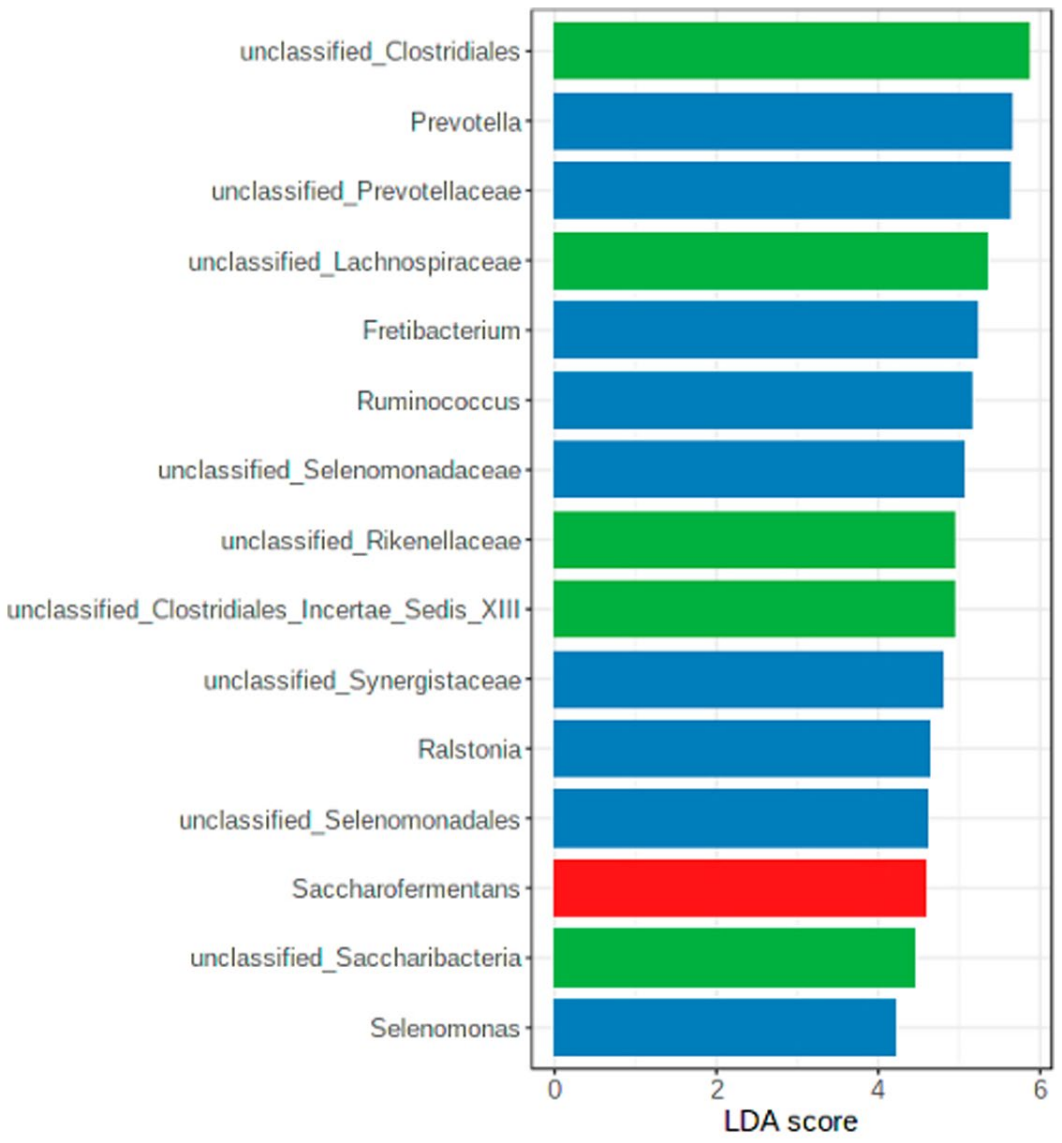
LEfSe diagram with the crucial genera per season (green = winter; blue = summer, red = spring).

A LEfSe was conducted to identify the differentially abundant genera among the seasons (Figure 6), confirming the results presented in Table 1.

**Figure 7:**
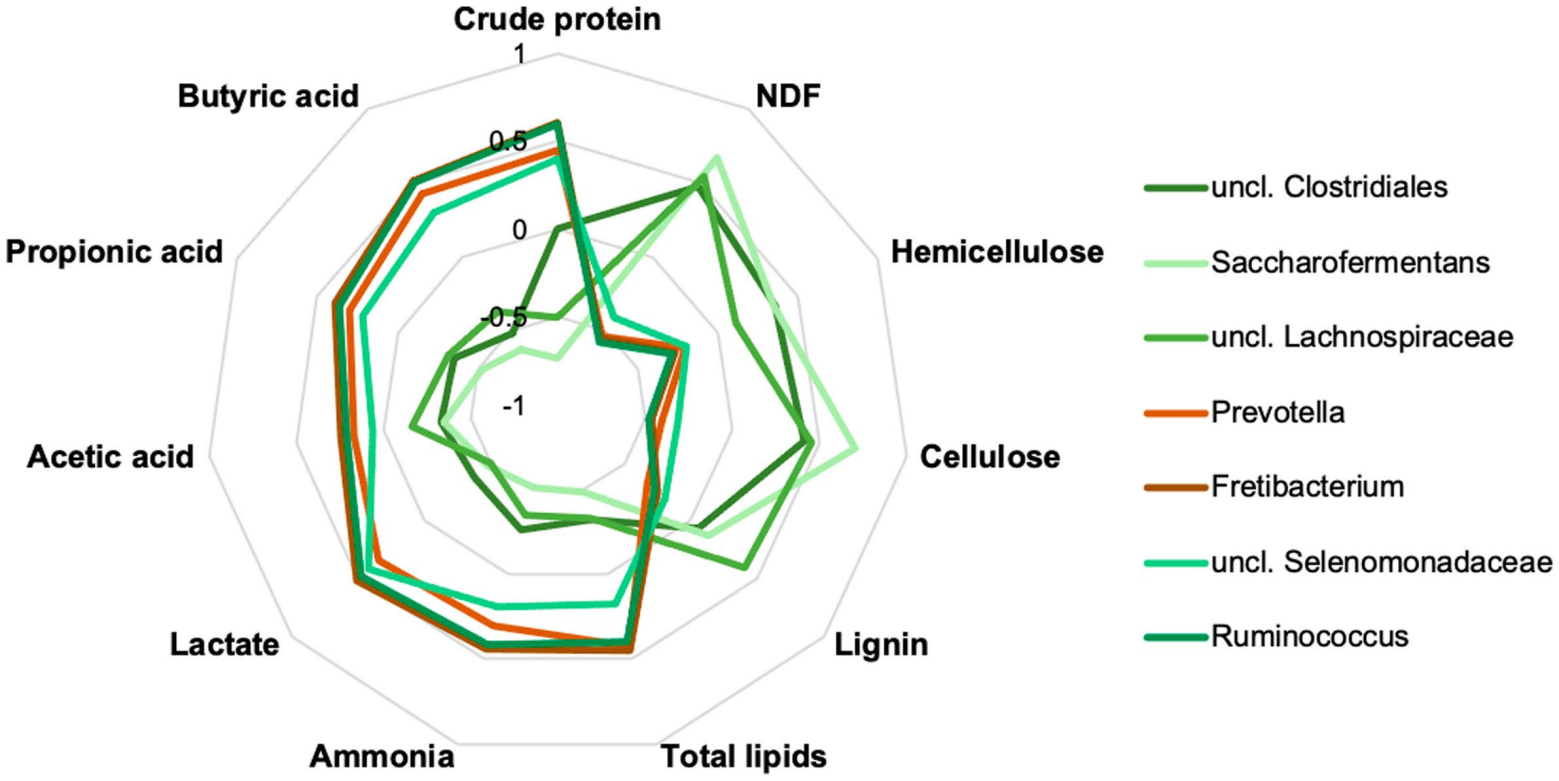
Correlation analyses showed significant positive and negative correlations of seven important rumen taxa to rumen content parameters. Data are provided in detail in Supplementary Table S6.

The top 15 genera were correlated with the crude nutrients and fermentation products (Figure 7; Supplementary Table S6). A group of three taxa, uncl. *Clostridiales*, uncl. *Lachnospiraceae* and *Saccharofermentans* were significantly positively correlated with NDF, hemicellulose, and cellulose, whereas significant negative correlations were identified with crude protein and various fermentation products. Opposite correlation results were obtained for *Prevotella*, *Fretibacterium*, uncl. *Selenomonadaceae* and *Ruminococcus*. Other taxa showed less or no significant correlations.

## 4. Discussion

The habitat of the chamois is strongly shaped by the season. The chamois have adapted passively and actively to changing environmental and climatic conditions and forage availability. This adaptation is reflected in the ingested plants, the resulting nutrient composition, and the rumen content’s adapted microbiota.

The highest proportion of mainly fresh grasses is consumed in summer, at around 80% (Supplementary Figure S1). In addition, more herbs and forbs are grazed. The crude protein and NFC content are highest in summer. When considering the crude protein content, it should always be noted that this also takes into account the microbial protein in the rumen content and not just that from ingested plants. In autumn, the shrubs of the plateaus play an important role alongside the grasses. Grasses are an essential part of the forage of the Bavarian chamois all year round, and on average, at least 56% of grasses can be found each season in the ingested forage. In winter, the proportion of conifers in the ingested forage increases substantially, and herbs & forbs are also consumed more frequently. The proportion of NDF and all fiber fractions significantly increased during this period. In spring, the proportion of plant categories consumed and the proportion of crude nutrients are average. This reflects the transition period between extreme winters and the start of the growing season.

However, the adaptations to the changing environment over the seasons are not only reflected in migratory movements and ingested food. It is well known that forage plays a vital role in the composition of the microbial community of wild ruminants, and this adapts accordingly (Henderson et al., 2015; Ricci et al., 2019; Li et al., 2022).

But, the knowledge about the microbiota of most wild animals still needs to be improved. For a few species, such as reindeer, there are now more studies available that have been carried out using genetic sequencing methods. In addition, some studies are currently known on ruminants from the Qinghai-Tibetian Plateau, which can be compared with the alpine chamois (Li et al., 2022). Comparability is also often problematic, as the number of samples is often minimal, or sample material originates from fecal or rumen samples with the limitation of fecal samples to improperly reflect the rumen bacterial populations. All studies have in common a high proportion of unclassified bacterial genera (Henderson et al., 2015). There are still many gaps in knowledge for wild animals, which also hampers the functional classifications of these microorganisms.

So far, one study from Italy is available for Apennine chamois (*R. pyrenaica ornate*, *n* = 5, fecal samples) (Smoglica et al., 2022). Species-specific differences were identified even at the phyla level. *Bacteroidetes* were detected with 48% as the dominant phylum by Smoglica et al. (2022), followed by *Firmicutes* with 39%. *Firmicutes* is known as the dominant phylum from other highland-bovids, such as the long-tailed gorals (Park et al., 2002), wild mountain goats (Haworth et al., 2019; Langda et al., 2020), takins (Chen et al., 2017), and from several African bovids (Nelson et al., 2003). In sheep of the Qinghai-Tibetian Plateau, *Firmicutes* dominate mainly in winter and *Bacteroidetes* in summer. In chamois, the phylum *Firmicutes* dominates throughout the year. Still, the proportion also increases strongly in winter (highest *Firmicutes/Bacteroidetes* rate), while *Bacteroidetes* increase strongly in summer (Fan et al., 2021). Similarities with other bovids can also be found at the genus level. High proportions of the genera *Ruminococcaceae, Prevotellaceae, Lachnospiraceae, Rikenellaceae*, and *Olsenella* are found in wild sheep and goats (Cunha et al., 2011; Haworth et al., 2019; Langda et al., 2020; Fan et al., 2021). Here, too, most genera are still unclassified. A significantly increased proportion of ruminococci in summer, as found in chamois, is also known from Tibetan sheep (Fan et al., 2021). Highland ruminants have been shown to have a more diverse microbiota than related lowland species (Wu et al., 2020; Li et al., 2022). Unfortunately, a closely related species from the alpine region is not available for comparison. But, apparent differences can be seen if we compare the chamois data with roe deer from the same alpine areas but from significantly lower altitudes (assuming the same analysis and filters). In contrast to the chamois, only 55 bacterial genera, which occur in larger numbers, were found in the rumen content of the roe deer, and 73 genera for the chamois. The average total VFA content is also significantly higher in the chamois, with an average of 10.5 mg/mL, compared to the roe deer with 6.7 mg/mL (Dahl et al., 27 April 2023, PREPRINT available at Research Square).^1^ The increased VFA content, which indicates increased microbial activity, is known from various species from the highlands (Zhang et al., 2016; Li et al., 2022). Generally, a strong influencing factor for the inhabitants of high altitudes is their exposure to strongly pronounced seasonal differences. This is reflected not only in chamois at the level of nutrients, microbiota, and their fermentation products. The microbiota of other highland species, such as yaks or Tibetan sheep, also seemed to react strongly to seasonal differences (Fan et al., 2021; Huang et al., 2022).

The genera uncl. *Clostridiales, Ruminococcaceae, Lachnospiracheae, Bacteroidales*, *Prevotella*, and *Ruminococcus* are part of the worldwide core microbiome of ruminants, defined by Henderson et al. (2015) also plays an essential role in the examined chamois. Many of these families contain fiber-degrading genera essential for converting plant material (Pitta et al., 2010; Biddle et al., 2013). For the uncl. *Clostridiales* and the uncl. *Lachnospiraceae* is this confirmed by a significant positive correlation with the content of NDF, cellulose, and lignin (see Figure 7). Both taxa correlated significantly negatively with the crude protein content. The high proportion of grasses in the forage causes a high proportion of NDF in the rumen. Outside the vegetation period, the animals consume more conifers and shrubs with a high total fiber content, specifically cellulose and lignin. The adaptation of the microbiota to very lignified plant parts becomes clear in autumn and winter (Figure 6), e.g., through other genera such as *Syntrophococcus*, which can demethylate lignin (Mosoni et al., 1994). Other examples are the taxa *Ruminococcaceae* or *Ruminococcus*, which have cellulolytic properties (Biddle et al., 2013).

*Prevotella and Prevotellaceae* are found in significantly high proportions in the rumen content in summer (see also Figure 6). These bacteria are described to be involved in the degradation of proteins and hemicellulose (Purushe et al., 2010). Our data showed that the proportion of crude protein is highest in the rumen content in summer, and in these months, most grasses are foraged, which contain high proportions of hemicellulose. The functional assignment is supported by the significantly positive correlation with crude proteins and the significantly negative correlation with cellulose and lignin (Figure 7). In addition, high crude protein contents lead to higher concentrations of ammonia and iso-branched short-chain fatty acids compared to values measured in autumn and winter. Thus, the higher abundance of *Prevotella* spp. in the first half of the year seems to be strongly linked to the availability of proteins and hemicellulose in the rumen.

Other top genera of the chamois with detected correlations are *Fretibacterium* and *Saccharofermentans -* the asaccharolytic genus *Fretibacterium* peaks (significantly) in summer (Naginyte et al., 2019). The significant positive correlation between proteins and propionic acid confirms the functional assignment to the medium and the fermentation product. The LEfSe (Figure 6) analysis underlines the importance of this genus, especially in the summer months when the protein content in the rumen is at its highest. *Saccharofermentans* is saccharolytic, significantly positively correlated with NDF, cellulose, and hemicellulose, and significantly negatively correlated with proteins (Figure 7). The degradation of cellulose is known from wetlands and could also occur in the rumen (Dai et al., 2016).

The genus *Olsenella* does not show clear correlations but is particularly common in the rumen content during summer; the asaccharolytic genus *Eggerthellaceae* (Gupta et al., 2013) has its lowest abundance here. Depending on the stage, grasses can contain significant amounts of water-soluble carbohydrates. The genus *Olsenella* can utilize mono-, di-, and polysaccharides (Kraatz et al., 2011; Zened et al., 2012; Chen et al., 2020).

Apart from the top 15 genera, the increased frequency of *Millionella* and *Paraeggerthella* is interesting. Both species were associated with high-fat diets and the promotion of obesity (Zhang et al., 2020). The plants available in the autumn and winter do not offer the chamois a high-fat content. Still, fat assimilation is extremely important in these months to draw on it in times of exceptionally high snow levels.

Genera such as *Denitrobacterium* and *Ralstonia* break down plant pathogens, xenobiotic compounds, and nitrotoxins (Anderson et al., 2005; Zhang and Qiu, 2016). *Sporobacter* is responsible for the degradation of aromatic compounds (Grech-Mora et al., 1996) and peaks in winter, which is probably due to enhanced lignin degradation.

Examples from the Pyrenees show that the chamois can also have a significantly different focus on specific foraging categories in other areas (Espunyes et al., 2019a). Here, the foraged key species in summer are, e.g., *Calluna vulgaris* and *Cystisus* spp., and forbs and grasses are included in significantly smaller proportions than other habitats. Even more extreme examples of chamois adaptation are known from New Zealand. If the chamois is not restricted to the alpine habitat, e.g., due to a lack of competition, it can very well use shrubs and hardwoods to a much greater extent. This is shown by a study where chamois mainly use forest areas at sea level and where the main diet in spring and summer consists of over 83% woody plants, and grasses only play a minor role (Yockney and Hickling, 2000). Woody plants will increasingly colonize the alpine grasslands during climate change (Espunyes et al., 2019b). The fact that chamois take advantage of a wide variety of food offers during the year without any long-term negative consequences for condition or reproduction can be considered a successful adaptation.

In addition to the bacterial microbiota, the microbial community is, of course, composed of other groups, such as protozoa or fungi. Since well-functioning primers are available for the bacteria and bacteria are known to be an essential part of the microbiome of wild ruminants, they have been the primary target. At least six protozoan species have been macroscopically described for chamois (Crha et al., 1985). The proportion of protozoa in the rumen is below the average of other wild ruminants (Clauss et al., 2011). There are even some studies that have found no protozoa at all in the rumen of some wild ruminant species or individuals (Drescher-Kaden and Seifelnasr, 1977; Ishaq and Wright, 2015). In any case, this offers a perspective for further research. An analysis of the anaerobic fungi should also be of great interest for wild ruminants, especially with regard to the degradation of lignin.

So, a future perspective will be to culture and classify more anaerobic species from the rumen of wild animals to better understand the functional assignments. Furthermore, other representatives of the microbial community, such as protozoa or fungi, should be identified, as these can also play an important role.

## 5. Conclusion

The 15 most important genera and other specific representatives of the chamois microbiota reflect its ability to adapt to its constantly changing and highly seasonal environment. The chamois in the alpine habitat can adapt excellently to the given forage availability and is a perfect example of the adaptability of wild ruminants to their environment. In addition to behavioral adaptations, such as horizontal and vertical migration movements, the anatomical and physiological requirements are also fulfilled to make use of a wide variety of forage categories. Insight into the interaction of the microbiota with crude nutrients and fermentation products has shown that, in parallel with physiological restructuring, the microbiota adapts very specifically to the given diet in order to achieve optimal energy gain. Chamois should have the adaptive capacity to cope successfully with climatic changes in terms of vegetation changes. And their adaptive microbiome plays an essential role in this.

## Data availability statement

The datasets presented in this study can be found in online repositories. The names of the repository/repositories and accession number(s) can be found at: https://www.ebi.ac.uk/ena, PRJEB62186.

## Ethics statement

Ethical approval was not required for the study involving animals in accordance with the local legislation and institutional requirements because, in Germany, chamois are considered a game species according to the Federal Hunting Act, paragraph 2, section 1 and are therefore ownerless. The chamois samples were obtained within the regular hunting bags during the regular hunting seasons and in the redevelopment areas for rehabilitating protection forests outside these because closed seasons had already been lifted, based on the ordinance on changing the hunting seasons for hoofed game in redevelopment areas in the administrative district of Upper Bavaria of 14 February 2014. The samples were provided to us by the employees of the participating forestry companies within the framework of our research project.

## Author contributions

S-AD, JS, AC-S, and AK contributed to the study conception and design. S-AD performed the material preparation and data collection. S-AD, AH-A, JS, and AC-S performed the data analysis. S-AD wrote the first draft of the manuscript. All authors commented on previous versions of the manuscript, and read and approved the final manuscript.

## Footnotes

^1^ https://doi.org/10.21203/rs.3.rs-2856341/v1
