# Supplementary material for: “Get the best out of what comes in” – adaptation of the microbiota of chamois (Rupicapra rupicapra) to seasonal forage availability in the Bavarian Alps
Source: Front Microbiol. 2023 Oct 2;14:1238744. doi: 10.3389/fmicb.2023.1238744 (PMC10577445; doi:10.3389/fmicb.2023.1238744)
Supplement: Supplementary file 1 [file Data_Sheet_1.pdf]

Supplementary Information

Table S1. Average content of crude nutrients in the dry matter of the rumen contents and fermentation products in the rumen liquid of chamois.

	Spring			Summer			Autumn			Winter		
	**Ø**	**sd**	**n**	**Ø**	**sd**	**n**	**Ø**	**sd**	**n**	**Ø**	**sd**	**n**
**Crude protein [%]**	21.82	5.97	13	23.60	2.75	13	18.36	3.53	14	14.64	2.98	8
**Total lipids [%]**	6.15	1.18	13	7.99	1.47	13	5.75	1.19	14	4.55	0.93	8
**Crude ash [%]**	6.28	1.21	13	7.65	0.74	13	6.82	0.96	14	6.05	0.52	8
**Non-fibre carbohydrates [%]**	10.22	3.24	13	13.00	4.47	13	12.04	3.26	13	10.46	2.46	8
**Neutral detergent fibre [%]**	55.53	9.21	13	47.76	4.08	13	58.16	7.31	14	64.30	3.66	8
**Acid detergent fibre [%]**	33.87	6.14	13	28.79	2.99	13	35.59	4.56	14	40.99	5.98	8
**Hemicellulose [%]**	21.66	5.73	13	18.97	4.65	13	22.58	6.34	14	23.31	2.73	8
**Cellulose [%]**	19.02	2.97	13	17.32	2.94	13	22.39	3.45	14	23.86	4.71	8
**Lignin [%]**	14.85	4.81	13	11.48	2.53	13	13.19	3.49	14	17.13	2.80	8
**Crude fibre [%]**	28.66	5.36	13	24.74	2.23	13	29.11	4.07	14	33.66	3.74	8
**Ammonia [mg/l]**	549.92	267.90	12	509.05	110.45	13	322.26	109.84	14	253.90	109.99	7
**Lactate [mg/l]**	429.28	215.69	11	572.00	222.00	13	412.00	223.00	14	251.00	85.00	6
**Acetic acid [mg/ml]**	5.83	1.10	12	6.02	0.86	13	5.55	1.14	14	4.75	0.96	7
**Propionic acid [mg/ml]**	2.33	0.65	12	2.38	0.49	13	1.95	0.44	14	1.79	0.59	7
**Butyric acid [mg/ml]**	1.68	0.46	12	1.90	0.36	13	1.43	0.37	14	1.04	0.29	7
**Valeric acid [mg/ml]**	0.25	0.09	12	0.22	0.05	13	0.16	0.05	14	0.15	0.07	7
**Isobutyric acid [mg/ml]**	0.14	0.05	12	0.12	0.03	13	0.09	0.02	14	0.07	0.02	7
**Isovaleric acid [mg/ml]**	0.31	0.09	11	0.21	0.08	13	0.21	0.08	13	0.20	0.14	7

Table S2. Results of the Kruskal-Wallis test to check the distribution of crude nutrients and fermentation products with regard to the factor season. Pairwise comparison with Bonferroni correction.

	**sig (two-tailed)**	**winter-autumn sig**	**winter-spring sig**	**winter-summer sig**	**autumn-spring sig**	**autumn-summer sig**	**spring-summer sig**
**Crude protein [%]**	<0.001	0.513	0.017	0	0.786	0.023	1
**Total lipids [%]**	<0.001	0.354	0.103	0	1	0.01	0.074
**Crude ash [%]**	0.002	0.811	1	0.008	0.963	0.26	0.005
**Non-fibre carbohydrates [%]**	0.139	1	1	0.513	1	1	0.198
**Neutral detergent fibre [%]**	<0.001	0.314	0.126	0	1	0.011	0.055
**Hemicellulose [%]**	0.202	1	1	0.331	1	0.73	0.671
**Cellulose [%]**	0.001	1	0.095	0.007	0.208	0.012	1
**Lignin [%]**	0.011	0.13	0.876	0.009	1	1	0.279
**Ammonia [mg/l]**	<0.001	1	0.026	0.003	0.103	0.01	1
**Lactate [mg/l]**	0.011	0.456	0.319	0.006	1	0.303	0.718
**Acetic acid [mg/ml]**	0.102	0.757	0.309	0.094	1	1	1
**Propionic acid [mg/ml]**	0.037	1	0.298	0.083	0.822	0.219	1
**Butyric acid [mg/ml]**	<0.001	0.508	0.029	0.001	1	0.042	1
**Valeric acid [mg/ml]**	0.007	1	0.076	0.178	0.042	0.12	1
**Isobutyric acid [mg/ml]**	0.002	0.959	0.003	0.027	0.062	0.467	1
**Isovaleric acid [mg/ml]**	0.02	1	0.071	1	0.052	1	0.079

Table S3. Number of reads analyzed for each sample and metadata.

Sample ID	Read count	Age class	Gender	Season	Sample ID	Read count	Age class	Gender	Season
Sample312	170144	subadult	male	autumn	Sample336	72708	juvenile	male	autumn
Sample313	170628	subadult	male	autumn	Sample337	58550	adult	female	autumn
Sample314	191292	adult	female	autumn	Sample338	55256	adult	male	autumn
Sample315	132276	adult	female	autumn	Sample339	63772	adult	male	summer
Sample316	161124	subadult	male	autumn	Sample340	67374	adult	male	spring
Sample317	196482	adult	female	winter	Sample341	101788	adult	male	spring
Sample318	151302	subadult	male	winter	Sample342	64708	adult	male	spring
Sample319	133400	adult	male	spring	Sample343	76940	adult	male	spring
Sample320	161896	juvenile	male	spring	Sample344	99738	juvenile	male	autumn
Sample321	174304	subadult	female	spring	Sample345	114370	adult	male	summer
Sample322	121864	adult	male	spring	Sample346	111720	adult	male	summer
Sample323	189100	subadult	male	spring	Sample347	77194	adult	female	summer
Sample324	133684	adult	female	winter	Sample348	75238	adult	male	summer
Sample325	85552	adult	male	autumn	Sample349	60398	adult	female	summer
Sample326	105700	adult	male	spring	Sample350	89654	adult	male	autumn
Sample327	65572	subadult	male	spring	Sample351	84022	adult	female	summer
Sample328	80510	juvenile	female	winter	Sample352	17024	adult	male	summer
Sample329	94690	juvenile	male	winter	Sample353	44992	adult	male	summer
Sample330	72310	adult	male	winter	Sample354	99576	adult	female	summer
Sample331	82490	adult	male	spring	Sample355	26562	adult	female	summer
Sample332	116286	juvenile	female	winter	Sample356	110998	adult	female	autumn
Sample333	52364	subadult	male	winter	Sample357	43338	adult	male	summer
Sample334	94300	subadult	male	autumn	Sample358	136798	adult	male	spring
Sample335	30470	juvenile	female	autumn	Sample359	137904	adult	male	summer

Botanical rumen content analysis

The analysis of the browsing was done by botanical rumen content analysis (BRCA). For this purpose, a part of the homogenized rumen content was rinsed with distilled water using wet sieving and three sieves with different mesh sizes (6.3, 3.55, and 1mm) (Onderscheka and Jordan, 1976; König et al., 2016). The macroscopically determinable parts were collected in sieves 1 and 2. In the third sieve, the smallest, macroscopically indeterminable solid components are collected. The determinable plant parts were macroscopically determined at genus name and, if possible, at the species level and assigned to defined browsing categories. They were then dried for 24 hours at 60 degrees Celsius and the percentage of the determinable mass was calculated.

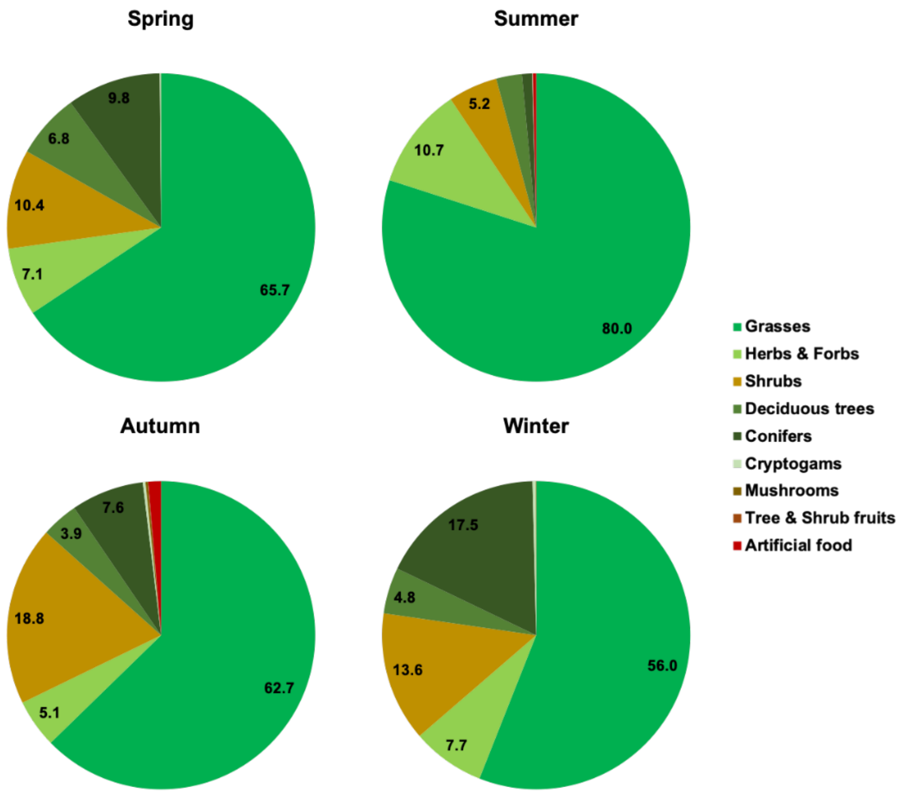

Figure S1. Average content of plant groups of the determinable plant mass in the rumen content per season [%].

Table S4. Relative abundance of bacterial phyla per season in the rumen content [%].

Phylum	Ø	sd	spring	sd	summer	sd	autumn	sd	winter	sd
Firmicutes	61.99	11.61	62.35	9.46	52.47	13.48	65.16	7.13	71.31	5.18
Bacteroidetes	19.79	11.63	18.14	8.72	28.63	14.59	17.25	7.46	12.55	7.05
Actinobacteria	13.97	6.59	15.26	6.24	12.24	9.68	14.35	4.14	13.98	2.59
Synergistetes	2.38	4.66	2.39	4.65	4.81	6.44	1.36	2.58	0.20	0.33
Proteobacteria	0.74	0.70	0.70	0.72	1.15	0.81	0.64	0.57	0.33	0.16
Candidatus Saccharibacteria	0.42	0.29	0.35	0.24	0.31	0.23	0.43	0.19	0.69	0.40
Spirochaetes	0.30	0.42	0.45	0.71	0.16	0.16	0.30	0.19	0.28	0.26
Tenericutes	0.06	0.07	0.03	0.04	0.02	0.02	0.09	0.08	0.11	0.11
Planctomycetes	0.03	0.05	0.04	0.07	0.00	0.01	0.03	0.04	0.05	0.05
Verrucomicrobia	0.03	0.06	0.01	0.01	0.07	0.10	0.01	0.01	0.01	0.01
SR1	0.01	0.02	0.00	0.01	0.01	0.01	0.01	0.02	0.04	0.04
**Uncl. *Bacteria***	0.17	0.13	0.14	0.07	0.08	0.04	0.26	0.14	0.22	0.17
**Uncl. *Saccharibacteria***	0.11	0.12	0.12	0.11	0.04	0.05	0.10	0.09	0.22	0.17

Table S5. Relative abundance of bacterial genera in the rumen content per season [%].

Genus	spring	sd	summer	sd	autumn	sd	winter	sd	average	sd
Actinomyces	0.01	0.01	0.00	0.00	0.01	0.01	0.01	0.01	0.01	0.01
Adlercreutzia	0.30	0.26	0.13	0.11	0.15	0.05	0.26	0.24	0.20	0.20
Aminicella	0.61	0.20	0.60	0.65	0.91	0.59	0.61	0.39	0.70	0.52
Anaerobutyricum	1.96	1.11	1.60	0.99	1.94	0.72	2.17	1.34	1.89	1.04
Anaerofustis	0.04	0.04	0.09	0.07	0.07	0.06	0.05	0.02	0.07	0.06
Beduinibacterium	0.04	0.07	0.01	0.02	0.05	0.05	0.05	0.07	0.04	0.06
Butyrivibrio	0.03	0.05	0.00	0.01	0.14	0.38	0.04	0.05	0.06	0.22
Clostridium IV	0.04	0.05	0.07	0.10	0.05	0.04	0.01	0.01	0.05	0.07
Coprococcus	0.30	0.20	0.37	0.27	0.55	0.40	0.43	0.20	0.41	0.31
Cutibacterium	0.02	0.06	0.03	0.04	0.02	0.03	0.00	0.00	0.02	0.04
Denitrobacterium	0.04	0.05	0.00	0.01	0.03	0.05	0.09	0.05	0.04	0.05

Duncaniella	0.22	0.31	0.60	0.85	0.45	0.45	0.20	0.19	0.39	0.56
Eggerthella	0.05	0.10	0.05	0.06	0.07	0.06	0.05	0.06	0.05	0.07
Enterococcus	0.00	0.01	0.03	0.05	0.05	0.10	0.00	0.00	0.03	0.06
Escherichia/Shigella	0.01	0.01	0.01	0.02	0.03	0.06	0.01	0.01	0.02	0.04
Fretibacterium	1.97	3.75	3.51	5.03	0.88	1.85	0.17	0.27	1.77	3.63
Ihubacter	0.17	0.19	0.06	0.05	0.09	0.06	0.10	0.06	0.11	0.12
Lachnospiracea incertae sedis	0.26	0.22	0.16	0.14	0.27	0.16	0.22	0.20	0.23	0.19
Lactobacillus	0.10	0.30	0.09	0.25	0.09	0.18	0.02	0.02	0.08	0.23
Lentimicrobium	0.01	0.02	0.08	0.23	0.01	0.02	0.02	0.03	0.03	0.13
Ligilactobacillus	0.09	0.10	0.19	0.27	0.40	0.70	0.03	0.02	0.20	0.43
Mediterranea	0.13	0.34	0.04	0.08	0.09	0.16	0.07	0.10	0.09	0.21
Millionella	0.03	0.05	0.00	0.01	0.03	0.04	0.03	0.02	0.02	0.04
Mitsuokella	0.15	0.32	0.23	0.32	0.08	0.11	0.01	0.01	0.13	0.25
Olsenella	5.36	2.59	5.40	4.64	4.17	1.33	4.29	1.99	4.85	3.02
Paraeggerthella	0.03	0.03	0.01	0.01	0.04	0.03	0.03	0.03	0.03	0.03
Paramuribaculum	0.01	0.01	0.00	0.00	0.01	0.02	0.03	0.03	0.01	0.02
Prevotella	5.95	6.64	10.41	7.53	4.10	2.22	1.49	0.99	5.87	6.22
Ralstonia	0.50	0.72	0.90	0.83	0.30	0.49	0.04	0.06	0.47	0.69
Ruminococcus	0.65	1.19	2.97	4.92	0.28	0.33	0.06	0.07	1.07	2.89
Saccharofermentans	1.24	1.12	0.47	0.66	1.22	0.60	1.18	0.31	1.02	0.83
Selenomonas	0.22	0.29	0.34	0.24	0.08	0.07	0.02	0.02	0.18	0.23
Slackia	0.21	0.13	0.16	0.14	0.28	0.11	0.32	0.19	0.24	0.15
Solobacterium	0.01	0.01	0.01	0.02	0.02	0.03	0.00	0.01	0.01	0.02
Sporobacter	0.03	0.04	0.01	0.01	0.04	0.05	0.09	0.06	0.04	0.05
Streptococcus	0.39	0.57	0.21	0.15	0.41	0.49	1.74	2.39	0.57	1.18
Succiniclasticum	0.07	0.22	0.01	0.01	0.01	0.01	0.02	0.02	0.03	0.12
Syntrophococcus	0.44	0.16	0.36	0.21	0.61	0.19	0.48	0.07	0.47	0.20
Treponema	0.45	0.71	0.16	0.16	0.30	0.19	0.28	0.26	0.30	0.42
unclassified Alphaproteobacteria	0.02	0.03	0.06	0.05	0.04	0.05	0.02	0.03	0.04	0.04
unclassified Atopobiaceae	0.77	0.41	0.60	0.45	0.95	0.41	0.88	0.37	0.79	0.44
unclassified Bacteria	0.14	0.07	0.08	0.04	0.26	0.14	0.22	0.17	0.17	0.13

unclassified Bacteroidales	7.07	4.59	6.52	2.79	5.72	2.69	5.67	2.73	6.29	3.39
unclassified Bacteroidetes	1.58	1.29	1.16	1.35	2.83	2.64	1.75	1.73	1.86	1.98
unclassified Bacteroidia	0.34	0.61	0.05	0.07	0.12	0.11	0.12	0.21	0.16	0.35
unclassified Christensenellaceae	0.30	0.20	0.29	0.24	0.29	0.17	0.39	0.22	0.31	0.21
unclassified Clostridia	0.01	0.01	0.00	0.01	0.01	0.01	0.03	0.07	0.01	0.03
unclassified Clostridiaceae 1	0.09	0.15	0.11	0.13	0.20	0.29	0.02	0.02	0.11	0.20
unclassified Clostridiales	29.35	11.61	20.92	10.61	28.62	4.65	35.37	6.16	27.86	10.14
unclassified Clostridiales Incertae Sedis XIII	2.40	0.77	0.85	0.45	2.18	0.60	2.53	0.81	1.94	0.94
unclassified Comamonadaceae	0.13	0.10	0.16	0.11	0.19	0.17	0.19	0.09	0.17	0.13
unclassified Coriobacteriia	1.05	0.52	0.62	0.50	0.91	0.44	0.72	0.40	0.84	0.50
unclassified Eggerthellaceae	7.42	2.95	5.25	4.19	7.72	2.65	7.33	2.24	6.90	3.32
unclassified Entomoplasmatales	0.02	0.02	0.01	0.02	0.05	0.06	0.09	0.09	0.04	0.06
unclassified Erysipelotrichaceae	0.27	0.18	0.33	0.32	0.47	0.25	0.40	0.30	0.37	0.28
unclassified Erysipelotrichia	0.04	0.02	0.03	0.04	0.04	0.03	0.04	0.03	0.04	0.03
unclassified Firmicutes	0.09	0.08	0.07	0.06	0.08	0.04	0.10	0.08	0.08	0.06
unclassified Lachnospiraceae	9.46	3.85	6.73	3.26	10.71	2.57	11.05	1.94	9.35	3.51
unclassified Mollicutes	0.02	0.03	0.01	0.01	0.04	0.05	0.02	0.02	0.02	0.03
unclassified Muribaculaceae	0.96	1.81	0.45	0.49	0.95	1.28	0.61	0.93	0.76	1.27
unclassified Negativicutes	0.44	0.97	0.18	0.16	0.22	0.33	0.44	0.94	0.31	0.67
unclassified Planctomycetes	0.04	0.07	0.00	0.01	0.03	0.04	0.05	0.05	0.03	0.05
unclassified Prevotellaceae	1.53	2.30	9.10	14.28	2.09	4.12	0.66	0.61	3.60	8.55
unclassified Proteobacteria	0.02	0.03	0.01	0.01	0.02	0.03	0.04	0.05	0.02	0.03
unclassified Rhizobiales	0.02	0.03	0.01	0.03	0.05	0.08	0.02	0.02	0.03	0.05
unclassified Rikenellaceae	0.31	0.33	0.20	0.37	0.85	0.87	1.90	1.51	0.70	1.01
unclassified Ruminococcaceae	11.25	3.40	11.60	3.27	13.07	1.99	13.19	4.64	12.20	3.39
unclassified Saccharibacteria	0.47	0.29	0.36	0.22	0.53	0.24	0.91	0.38	0.53	0.33
unclassified Selenomonadaceae	1.15	1.30	2.57	2.87	1.20	1.38	0.33	0.20	1.41	1.96
unclassified Selenomonadales	0.66	0.81	0.90	0.83	0.68	1.16	0.10	0.10	0.64	0.91
unclassified SR1 genera incertae sedis	0.00	0.01	0.01	0.01	0.01	0.02	0.04	0.04	0.01	0.02
unclassified Subdivision5	0.01	0.01	0.07	0.10	0.01	0.01	0.01	0.01	0.03	0.06
unclassified Synergistaceae	0.42	0.95	1.30	1.77	0.48	0.93	0.03	0.06	0.61	1.24

Table S6. Correlations (Spearman-Rho) between the top 15 bacterial genera and the nutrients and fermentation products. (cc = correlation coefficient, sig = significance (two-tailed)). * The correlation is significant at the 0.05 level. ** The correlation is significant at the 0.01 level. Significant correlations are marked in bold.

	uncl. Clostridiales		***uncl. Ruminococcaceae***		***uncl. Lachnospiraceae***		***uncl. Eggerthellaceae***		***uncl. Bacteroidales***		***Prevotella***		***Olsenella***		***uncl. Prevotellaceae***	
	cc	sig	cc	sig	cc	sig	cc	sig	cc	sig	cc	sig	cc	sig	cc	sig
**Crude protein**	**-.456****	**0.001**	-0.218	0.136	**-.499****	**0.000**	-0.211	0.149	-0.157	0.288	**.446****	**0.002**	0.080	0.587	**.338***	**0.019**
**NFC**	-0.224	0.130	0.024	0.871	-0.172	0.248	-0.021	0.888	0.032	0.830	0.248	0.093	0.036	0.810	0.286	0.051
**NDF**	**.479****	**0.001**	0.123	0.406	**.541****	**0.000**	0.189	0.199	0.124	0.401	**-.536****	**0.000**	-0.075	0.613	**-.458****	**0.001**
**Hemicellulose**	**.361***	**0.012**	**-.288***	**0.047**	0.115	0.435	-0.071	0.629	0.094	0.525	-0.207	0.159	-0.040	0.788	-0.110	0.455
**Cellulose**	**.417****	**0.003**	0.221	0.131	**.461****	**0.001**	0.220	0.134	-0.065	0.661	**-.406****	**0.004**	-0.154	0.295	**-.323***	**0.025**
**Lignin**	0.060	0.684	0.219	0.136	**.401****	**0.005**	0.150	0.308	0.149	0.313	**-.324***	**0.025**	0.082	0.582	**-.345***	**0.016**
**Total lipids**	**-.325***	**0.024**	-0.084	0.568	**-.332***	**0.021**	-0.031	0.837	-0.047	0.749	**.424****	**0.003**	0.198	0.177	0.226	0.122
**Crude ash**	**-.361***	**0.012**	0.106	0.472	**-.353***	**0.014**	-0.186	0.206	0.039	0.795	0.262	0.072	-0.127	0.388	0.272	0.062
**Ammonia**	-0.268	0.072	-0.166	0.270	**-.353***	**0.016**	-0.026	0.863	-0.218	0.145	**.306***	**0.039**	0.098	0.519	0.115	0.448
**Lactate**	**-.374***	**0.012**	0.117	0.451	**-.497****	**0.001**	-0.280	0.066	-0.033	0.834	**.342***	**0.023**	-0.173	0.262	**.308***	**0.042**
**Acetic acid**	**-.331***	**0.025**	-0.144	0.339	-0.164	0.277	0.128	0.398	-0.010	0.949	0.167	0.269	0.282	0.058	0.116	0.441
**Propionic acid**	**-.353***	**0.016**	-0.150	0.320	**-.320***	**0.030**	-0.081	0.591	-0.040	0.794	**.300***	**0.043**	0.146	0.331	0.233	0.119
**Isobutyric acid**	-0.168	0.265	-0.239	0.110	-0.217	0.148	0.014	0.925	0.022	0.886	0.235	0.116	0.275	0.065	0.064	0.673
**Butyric acid**	**-.516****	**0.000**	-0.136	0.368	**-.365***	**0.013**	-0.113	0.456	-0.079	0.602	**.428****	**0.003**	0.097	0.520	0.285	0.055
**Isovaleric acid**	-0.115	0.458	-0.293	0.054	-0.077	0.619	0.064	0.680	-0.030	0.846	0.065	0.676	**.368***	**0.014**	0.011	0.942
**Valeric acid**	-0.259	0.083	-0.216	0.150	-0.272	0.067	-0.052	0.732	-0.012	0.935	0.193	0.199	0.104	0.490	0.068	0.652

	uncl. Clostridiales Incertae Sedis XIII		*Anaerobutyricum*		*uncl. Bacteroidetes*		*Fretibacterium*		*uncl. Selenomonadaceae*		*Ruminococcus*		*Saccharofermentans*	
	cc	sig	cc	sig	cc	sig	cc	sig	cc	sig	cc	sig	cc	sig
**Crude protein**	**-.395****	**0.005**	-0.074	0.618	-0.209	0.154	**.607****	**0.000**	**.400****	**0.005**	**.594****	**0.000**	**-.732****	**0.000**
**NFC**	-0.257	0.081	-0.228	0.123	0.085	0.571	0.049	0.744	0.113	0.450	0.182	0.220	-0.160	0.282
**NDF**	**.500****	**0.000**	0.188	0.200	0.193	0.189	**-.543****	**0.000**	**-.405****	**0.004**	**-.569****	**0.000**	**.672****	**0.000**
**Hemicellulose**	0.255	0.081	0.170	0.248	0.222	0.130	-0.264	0.070	-0.199	0.175	**-.286***	**0.049**	**.353***	**0.014**
**Cellulose**	**.380****	**0.008**	-0.111	0.451	0.098	0.506	**-.462****	**0.001**	**-.312***	**0.031**	**-.476****	**0.001**	**.704****	**0.000**
**Lignin**	0.252	0.085	**.345***	**0.016**	0.067	0.650	-0.252	0.084	-0.194	0.186	-0.273	0.060	0.130	0.377
**Total lipids**	-.442**	0.002	-0.069	0.643	-0.107	0.471	**.453****	**0.001**	0.170	0.247	**.395****	**0.005**	**-.489****	**0.000**
**Crude ash**	**-.309***	**0.033**	**-.324***	**0.025**	**-.386****	**0.007**	**.422****	**0.003**	**.285***	**0.050**	**.486****	**0.000**	**-.300***	**0.038**
**Ammonia**	-0.218	0.146	-0.143	0.345	-0.210	0.160	**.441****	**0.002**	0.195	0.194	**.413****	**0.004**	**-.515****	**0.000**
**Lactate**	**-.307***	**0.043**	-0.050	0.749	-0.222	0.148	**.512****	**0.000**	**.420****	**0.005**	**.474****	**0.001**	**-.474****	**0.001**
**Acetic acid**	-0.162	0.281	-0.126	0.406	-0.018	0.904	0.242	0.105	0.057	0.706	0.214	0.154	**-.350***	**0.017**
**Propionic acid**	-0.283	0.057	-0.053	0.726	-0.215	0.152	**.394****	**0.007**	0.220	0.142	**.360***	**0.014**	**-.522****	**0.000**
**Isobutyric acid**	-0.078	0.608	-0.004	0.981	-0.025	0.872	0.221	0.139	0.074	0.627	0.159	0.292	**-.334***	**0.023**
**Butyric acid**	**-.413****	**0.004**	-0.116	0.442	-0.124	0.412	**.513****	**0.000**	**.300***	**0.043**	**.505****	**0.000**	**-.619****	**0.000**
**Isovaleric acid**	0.042	0.789	**.312***	**0.040**	-0.013	0.933	0.203	0.186	0.040	0.795	0.063	0.686	-0.224	0.143
**Valeric acid**	-0.111	0.463	-0.118	0.434	-0.151	0.318	**.334***	**0.023**	0.189	0.208	0.290	0.051	**-.482****	**0.001**
